# Supplementary material for: ICAN: Interpretable cross-attention network for identifying drug and target protein interactions
Source: PLoS One. 2022 Oct 24;17(10):e0276609. doi: 10.1371/journal.pone.0276609 (PMC9591068; doi:10.1371/journal.pone.0276609)
Supplement: S2 Fig — (PDF) [file pone.0276609.s002.pdf]

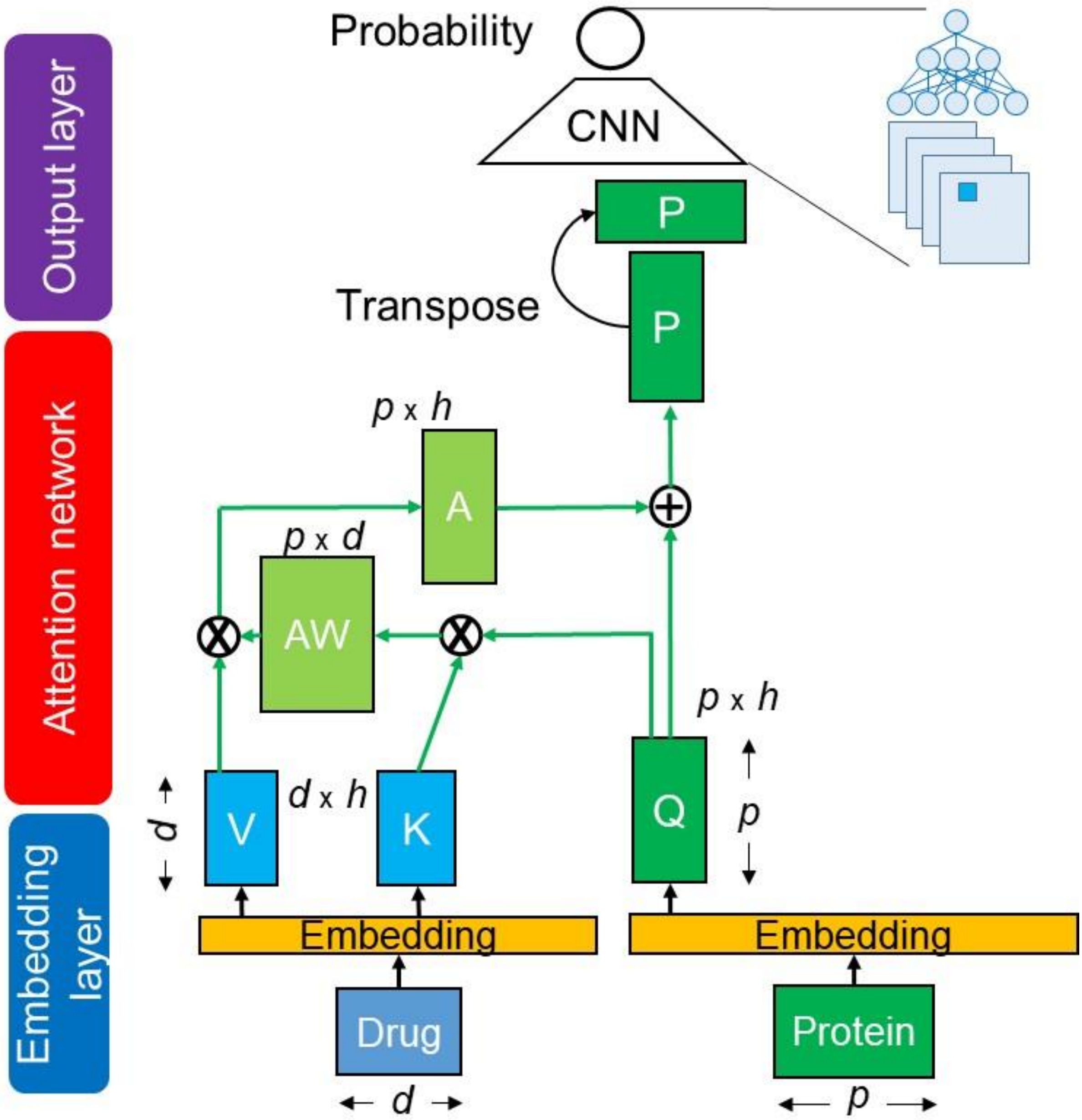

Figure S2 Cross-attention–based model

This network corresponds to CA_P with nn.Embedding of FCS (Table 3). It uses only protein context features. Q, K, and V denote Query, Key, and Value matrixes.

AW: attention-weight matrix, A: attention matrix, D: drug-context matrix, P: protein-context matrix, d: length of drug sequence, p: length of protein sequence, h: hidden dimension size.
