# Supplementary material for: ICAN: Interpretable cross-attention network for identifying drug and target protein interactions
Source: PLoS One. 2022 Oct 24;17(10):e0276609. doi: 10.1371/journal.pone.0276609 (PMC9591068; doi:10.1371/journal.pone.0276609)
Supplement: S1 Table — (PDF) [file pone.0276609.s003.pdf]

Table S1 Performance of different network architectures

Architecture		SN	SP	ROCAUC	PR	F1	PRAUC
CA_DP_FCL	Mean	0.802	**0.814**	0.890	**0.186**	**0.302**	0.359
	Std	0.012	0.009	0.004	0.007	0.009	0.017
CA_DP	Mean	0.843	0.789	0.897	0.183	0.299	0.367
	Std	0.044	0.066	0.006	0.038	0.048	0.016
CA2_DP	Mean	0.826	0.793	0.884	0.179	0.293	0.314
	Std	0.044	0.043	0.010	0.025	0.031	0.011
CA3_DP	Mean	0.833	0.777	0.880	0.166	0.277	0.284
	Std	0.029	0.019	0.007	0.009	0.012	0.036
SA_DP	Mean	0.818	0.710	0.840	0.131	0.226	0.243
	Std	0.034	0.040	0.008	0.012	0.017	0.013
CA_D	Mean	0.799	0.812	0.879	0.186	0.301	0.306
	Std	0.023	0.026	0.008	0.017	0.021	0.014
CA_P (ICAN)	Mean	**0.884**	0.766	**0.903**	0.167	0.281	**0.372**
	Std	0.011	0.016	0.005	0.009	0.012	0.032

PR denotes precision. F1 denotes F1-score that is the harmonic mean of PR and recall (SP). Mean and Std denote the mean and standard deviation of each metric. The architectures are shown in Table 3. Bold values indicate the best-performing method for each metric.
