# Supplementary material for: ICAN: Interpretable cross-attention network for identifying drug and target protein interactions
Source: PLoS One. 2022 Oct 24;17(10):e0276609. doi: 10.1371/journal.pone.0276609 (PMC9591068; doi:10.1371/journal.pone.0276609)
Supplement: S2 Table — (PDF) [file pone.0276609.s004.pdf]

Table S2 Performance of different encoding methods in CA_P (ICAN)

Encoding method		SN	SP	ROCAUC	PR	F1	PRAUC
nn.Embedding of FCS	Mean	**0.884**	0.766	**0.903**	0.167	0.281	**0.372**
	Std	0.011	0.016	0.005	0.009	0.012	0.032
nn.Embedding of SMILES	Mean	0.817	0.810	0.888	**0.189**	**0.306**	0.343
	Std	0.044	0.039	0.014	0.026	0.033	0.033
nn.Embedding of SELFIES	Mean	0.798	**0.815**	0.889	0.188	0.304	0.357
	Std	0.045	0.029	0.008	0.014	0.016	0.026
One-hot encoding of SMILES	Mean	0.424	0.802	0.692	0.063	0.109	0.135
	Std	0.404	0.194	0.101	0.060	0.104	0.064
One-hot encoding of SELFIES	Mean	0.776	0.724	0.832	0.151	0.246	0.225
	Std	0.080	0.142	0.034	0.059	0.078	0.041

PR denotes precision. F1 denotes F1-score that is the harmonic mean of PR and recall (SP). Mean and Std denote the mean and standard deviation of each metric. The encoding methods are shown in Table 2. Bold values indicate the best-performing method for each metric.
