# Supplementary material for: ICAN: Interpretable cross-attention network for identifying drug and target protein interactions
Source: PLoS One. 2022 Oct 24;17(10):e0276609. doi: 10.1371/journal.pone.0276609 (PMC9591068; doi:10.1371/journal.pone.0276609)
Supplement: S3 Table — (PDF) [file pone.0276609.s005.pdf]

Table S3 Performance of different learning methods on the DAVIS test dataset

Method		SN	SP	ROCAUC	PR	F1	PRAUC
LR	Mean	0.699	0.842	0.835	-	-	0.232
	Std	0.051	0.033	0.010	-	-	0.023
GNN-CPI	Mean	0.696	0.842	0.840	-	-	0.269
	Std	0.047	0.039	0.012	-	-	0.020
DeepDTI	Mean	0.751	**0.853**	0.861	-	-	0.231
	Std	0.015	0.012	0.002	-	-	0.006
DeepDTA	Mean	0.878	0.711	0.879	0.140	0.242	0.284
	Std	0.023	0.040	0.008	0.013	0.019	0.022
DeepConv-DTI	Mean	0.835	0.794	0.890	0.180	0.295	0.341
	Std	0.036	0.039	0.014	0.023	0.032	0.041
TransformerCPI	Mean	0.801	0.728	0.831	0.135	0.231	0.202
	Std	0.023	0.020	0.006	0.006	0.009	0.007
MolTrans	Mean	0.857	0.800	0.901	**0.185**	**0.304**	0.361
	Std	0.003	0.001	0.001	0.002	0.002	0.003
CA_P (ICAN)	Mean	**0.884**	0.766	**0.903**	0.167	0.281	**0.372**
	Std	0.011	0.016	0.005	0.009	0.012	0.032

PR denotes precision. F1 denotes F1-score that is the harmonic mean of PR and recall (SP). Mean and Std denote the mean and standard deviation of each metric. Bold values indicate the best-performing method for each metric.
