# Supplementary material for: ICAN: Interpretable cross-attention network for identifying drug and target protein interactions
Source: PLoS One. 2022 Oct 24;17(10):e0276609. doi: 10.1371/journal.pone.0276609 (PMC9591068; doi:10.1371/journal.pone.0276609)
Supplement: S4 Table — (PDF) [file pone.0276609.s006.pdf]

**Table S4 Performance of different learning methods on the BindingDB test dataset**

| Method | | SN | SP | ROCAUC | PR | F1 | PRAUC |
|---|---|---|---|---|---|---|---|
| LR | Mean | 0.741 | 0.896 | 0.887 | - | - | 0.557 |
| | Std | 0.013 | 0.011 | 0.002 | - | - | 0.015 |
| GNN-CPI | Mean | 0.754 | **0.903** | 0.900 | - | - | 0.578 |
| | Std | 0.015 | 0.011 | 0.004 | - | - | 0.015 |
| DeepDTI | Mean | 0.651 | 0.895 | 0.844 | - | - | 0.429 |
| | Std | 0.024 | 0.023 | 0.002 | - | - | 0.005 |
| DeepDTA | Mean | **0.907** | 0.749 | 0.898 | 0.385 | 0.537 | 0.587 |
| | Std | 0.043 | 0.070 | 0.034 | 0.054 | 0.047 | 0.132 |
| DeepConv-DTI | Mean | **0.907** | 0.749 | 0.898 | 0.385 | 0.537 | 0.587 |
| | Std | 0.043 | 0.070 | 0.034 | 0.054 | 0.047 | 0.132 |
| TransformerCPI | Mean | 0.855 | 0.782 | 0.886 | 0.398 | 0.542 | 0.544 |
| | Std | 0.021 | 0.028 | 0.002 | 0.022 | 0.018 | 0.008 |
| MolTrans | Mean | 0.845 | 0.834 | **0.906** | **0.462** | **0.597** | 0.590 |
| | Std | 0.006 | 0.014 | 0.003 | 0.019 | 0.015 | 0.005 |
| CA_P (ICAN) | Mean | 0.846 | 0.815 | 0.900 | 0.434 | 0.574 | **0.604** |
| | Std | 0.023 | 0.015 | 0.003 | 0.014 | 0.008 | 0.016 |

PR denotes precision. F1 denotes F1-score that is the harmonic mean of PR and recall (SP). Mean and Std denote the mean and standard deviation of each metric. Bold values indicate the best-performing method for each metric.
