# Supplementary material for: ICAN: Interpretable cross-attention network for identifying drug and target protein interactions
Source: PLoS One. 2022 Oct 24;17(10):e0276609. doi: 10.1371/journal.pone.0276609 (PMC9591068; doi:10.1371/journal.pone.0276609)
Supplement: S5 Table — (PDF) [file pone.0276609.s007.pdf]

Table S5 Performance of different learning methods on the BIOSNAP test dataset

Method		SN	SP	ROCAUC	PR	F1	PRAUC
LR	Mean	0.755	0.800	0.846	-	-	0.850
	Std	0.039	0.018	0.004	-	-	0.011
GNN-CPI	Mean	0.780	0.819	0.879	-	-	0.890
	Std	0.014	0.012	0.007	-	-	0.004
DeepDTI	Mean	0.789	**0.845**	0.876	-	-	0.876
	Std	0.027	0.017	0.005	-	-	0.006
DeepDTA	Mean	0.826	0.779	**0.888**	**0.797**	0.809	**0.895**
	Std	0.051	0.088	0.006	0.051	0.012	0.005
DeepConv-DTI	Mean	0.821	0.755	0.881	0.780	0.796	0.891
	Std	0.081	0.095	0.006	0.055	0.018	0.007
TransformerCPI	Mean	**0.848**	0.773	0.880	0.791	**0.818**	0.880
	Std	0.003	0.008	0.002	0.006	0.003	0.003
MolTrans	Mean	0.820	0.780	0.881	0.791	0.805	0.892
	Std	0.001	0.000	0.000	0.000	0.000	0.000
CA_P (ICAN)	Mean	0.799	0.786	0.871	0.791	0.795	0.886
	Std	0.012	0.018	0.001	0.012	0.003	0.002

PR denotes precision. F1 denotes F1-score that is the harmonic mean of PR and recall (SP). Mean and Std denote the mean and standard deviation of each metric. Bold values indicate the best-performing method for each metric.
